# Observations on the Treatment of Convulsions during Parturition

**Published:** 1781-11

**Authors:** 


### SECTION II.

### ESSAYS AND OBSERVATIONS.

I. *Obſervations on the treatment of Convulſions during parturition. By* Robert Bland, *M. D. Phyſician Man-midwife to the Weſtminſter General Diſpenſary. Read October* 15, 1781.

IN the courſe of my attendance on the midwifery department of the Weſtminſter General Diſpenſary, four caſes of convulſion during parturition have fallen under my care. As this is by no means a frequent accident, and as the cauſe of it ſeems to be generally miſunderſtood, I flatter myſelf that a ſhort account of the caſes, with with ſome obſervations on the treatment of convulſions at that period, may not be unacceptable to the Society.

The firſt of the patients was a young woman, who in the 7th month of her firſt pregnancy, ſuddenly became convulſed, and continued to be ſo at intervals for four days. The firſt fit laſted half an hour; the ſubſequent ones were of ſhorter duration; and at length, by repeated blood-letting, and the uſe of purgative and anodyne medicines, they were ſubdued, and at the end of three weeks more, by an eaſy and expeditious labour, the woman was delivered of a child, which, from its appearance, had probably been dead three or four weeks. In two other women the convulſions did not begin till the labour was pretty far advanced. The ſame methods were adopted in theſe caſes as in the one juſt now mentioned, but without any good effect, as the ſpaſms continued till the patients were delivered, after which they ceaſed, and the women recovered without any intervening accident. The children were born alive; and in both caſes the delivery was apparently accelerated by the convulſions.—Of the fourth caſe, which occurred ſince I thought of communicating this paper to the Society, I ſhall give a more particular account. The patient was a young woman, twenty-four years of age, ſhort of ſtature, and of a delicate habit. On Friday the 14th of September laſt, being near her full time, ſhe was taken with labour pains, attended with a mucous diſcharge from the uterus, the entrance to which was a little dilated. The pains continued, but with long intervals, till Wedneſday evening, (Sept. 19) when ſhe was ſeized with convulſions. During the fit, ſhe was ſenſeleſs, and foamed at the mouth, her head at the ſame time being drawn over her ſhoulder, her countenance diſtorted, and her whole frame extremely agitated. She remained in this ſtate forty minutes, when ſhe fell aſleep, and after awaking at the end of half an hour, appeared at firſt ſomewhat wild and incoherent, but ſoon recovered herſelf. It was at this period of the caſe that I firſt ſaw the patient. She had now frequent returns of ſlight pains, her ſkin felt hot, and her pulſe beat with conſiderable quickneſs. She was coſtive, but voided her urine freely. The os uteri, which was dilated to the ſize of a half-crown piece, was thick, rigid, and of a conical ſhape. The head of the child reſted above the brim of the pelvis.

With a view to prevent a return of the ſpaſms, I directed eight ounces of blood to be taken from the the arm, but this was inſtantly productive of a ſecond convulſive paroxyſm, as formidable in every reſpect as the firſt, and of equal duration. Like that, it terminated in a ſound ſleep. When the patient awaked, a common glyſter was injected, and procured a plentiful ſtool. About an hour afterwards, thirty drops of the *tinctura thebaica* were adminiſtered, by means of which ſhe ſlept quietly during the remainder of the night; but at ſix the next morning, (Sept. 20) ſhe had a third convulſive fit of nearly the ſame violence as the preceding ones. Another glyſter was now thrown up, but this producing no effect, twelve grains of jalap and four of calomel were exhibited, and to quicken their operation, three or four ſpoonfuls of a ſolution of purging ſalts were directed to be taken every three or four hours, till the bowels ſhould be ſufficiently emptied.

The patient had no return either of convulſions or labour-pains, and by repeating the opiate in the evening, ſhe reſted well during the night. The next morning I found her ſkin cool and ſoft, her pulſe of its natural quickneſs, and her countenance cheerful. She was deſirous of drinking a little porter, which was allowed her; and as ſhe had been ſufficiently purged, the opiate was repeated at night. At ſix the next morning

 (Sept.

(Sept. 21) the labour-pains re-commenced, and at ten ſhe was delivered of a child, which ſeemed to have been dead ſeveral days. The placenta not being detached at the expiration of two or three hours, I was again ſent for by the midwife who attended the patient, and although I was obliged to introduce my hand into the uterus, and to exert ſome force in ſeparating the cake, it did not in the leaſt degree renew the convulſions.

For the production of convulſions during child-birth, various cauſes have been aſſigned, ſuch as the death of the child *in utero*, fullneſs of the blood-veſſels, extreme irritability or inflammation of the *os uteri*, cloſe hot rooms, cordials, &c. but neither of theſe ſuppoſed cauſes ſatisfactorily account for them, it being well known that the greater number of women ſimilarly circumſtanced eſcape, while others of different and oppoſite temperaments and ſituations, fall a ſacrifice to them. Thus many women bear dead children within them ſeveral weeks, without ever experiencing convulſions; and thin and delicate women are found to be as ſubject to them as the plethoric and robuſt. Again, women who are ſo irritable, that they can hardly bear the ſlighteſt handling of the *os uteri*, eſcape them; as do others alſo, who have ſuffered laceration of the vagina, or or other local injury in parturition. Laftly, if clofe hot rooms and cordials could induce them, we know that at one time at leaft, they muft have been very frequent; which is not the cafe. But if we reject the agency of the feveral caufes we have enumerated, ftill lefs fhall we be inclined to admit that of the labour-pains, as being able either to excite or to continue them; not only becaufe fo very large a number of child-bearing women are exempt from them, but becaufe women whofe labours are peculiarly fevere, are not more fubject to them than thofe who have the eafieft births; and becaufe convulfions do not neceffarily and conftantly terminate with the delivery of the child. The opinion that convulfions are occafioned, or at leaft continued by the labour-pains, probably took its rife from its having been obferved that they frequently ceafed on the birth of the child; but this ceafing of convulfions at that period, when it does happen, arifes from another caufe, and when the labour is too precipitately terminated, is frequently fucceeded by the moft dangerous and fatal fymptoms, as will be explained by and by. It will in general, I believe, be equally vain to attempt to fearch for the caufe of them in the affections of the mind; for although any great affliction, or fudden and violent violent guft of paffion, may be fuppofed fufficient to produce them, they will oftener be found where no fenfible difturbance has preceded.— *Dr. Denman feems to think they are almoft peculiar to great towns, where people live luxurioufly; but the circumftances of the fubjects of thefe obfervations do not corroborate that conjecture: they were all amongft the lower clafs of people, and had little opportunity of injuring themfelves by indolence or intemperance; neither could I learn any circumftance previous to the appearance of convulfions, in any of thefe cafes, which would have led to the knowledge of their approach, and confequently pointed out any method of preventing them. From thefe reflections, it would feem that convulfions in this ftate have nothing peculiar in their caufe from thofe, which happen to perfons differently fituated; and although external agents, particularly violent affections of the mind, may fometimes, as at other periods, excite them; yet this will rarely happen, unlefs there is fome peculiar vice in the conftitution, difpofing to them: and from obfervation I think we are juftified in faying, that the puerperal ftate is far from favouring the irruption of them; as women at that time may do and fuffer, almoft

* Effay on Puerperal Convulfions, 1768.

with with impunity, what at any other time would be attended with the moſt ſerious conſequences. But whatever may be the cauſe, there is evidently in the fit, as in the apoplexy, a too rapid and dangerous determination of the blood to the head, which demands the moſt immediate and ſerious attention. To remedy this, blood muſt be immediately drawn, and, if poſſible, from the jugulars. The ſtate of the labour ſhould then be inquired into, and if the child is not too far advanced in the pelvis, it will be right to preſcribe a large ſtimulating glyſter to empty the bowels, and at the ſame time leſſen the determination to the head; this, if not ſufficient for the purpoſe, ſhould be aſſiſted by a few grains of jalap and calomel, or ſome other briſk purge. If the labour is far advanced, the convulſions will act upon the fœtus; and if there is no impediment, either from wrong preſentation, or diſproportion of the child to the pelvis, will in a little time ſafely expel it. If there ſhould be found to be any obſtacle to the delivery, the poſition of the child, if faulty, muſt be altered; or we ought to have recourſe to other neceſſary aſſiſtance, in the ſame manner as if convulſions were not preſent. In either way the termination of the labour will frequently put an end to the convulſions. But if this is too haſtily haftily performed, before the veffels have been properly emptied, and the rapid motion of the blood in them diminifhed, there will be danger, from the torrent rufhing too impetuoufly into the inteftines, or other abdominal vifcera, of inflammation in fome of thofe parts inducing puerperal fever, and oftentimes death. But where the labour is not far advanced, after the exhibition of the glyfter and purgative, thirty drops of the tinctura thebaica may be given and repeated, interpofing occafionally the glyfter or cathartic as fymptoms fhall indicate. I have faid nothing of the ufe of bathing or fomenting the feet and legs, both becaufe I have had no experience of their efficacy in thefe cafes, and becaufe, until other methods by evacuations, &c. have been tried, they feem to me by their effect of increafing the volume of the blood, to have a tendency rather to increafe than diminifh the fpafms.

I have now finifhed the tafk I impofed upon myfelf of inquiring into the nature of what are called (I think improperly) puerperal convulfions, and I have endeavoured to overturn an opinion concerning them, which feemed to tend to the introduction of a practice highly dangerous to the patient. If my reafoning fhould be thought juft, and the practical cautions relative to to too hasty delivery in this case salutary, I shall have the heart-felt satisfaction of believing, that I may one day be instrumental in saving the life of one or more objects, about to be sacrificed by too close an adherence to a rule, taken up, I believe, too precipitately, and without sufficient examination.

